# Variation of Marginal Mandibular Nerve in a Caucasian Male Cadaver: A Study Using the Anatomage Table

**DOI:** 10.7759/cureus.6168

**Published:** 2019-11-16

**Authors:** Paschalis Strantzias, Anna Botou, Arezina Manoli, Panagiotis N Skandalakis, Dimitrios Filippou

**Affiliations:** 1 Oral Medicine, Medical School of National and Kapodistrian University of Athens, Athens, GRC; 2 Ophthalmology, Medical School of National and Kapodistrian University of Athens, Athens, GRC; 3 Otolaryngology, Medical School of National and Kapodistrian University of Athens, Athens, GRC; 4 Surgery, Medical School of National and Kapodistrian University of Athens, Athens, GRC

**Keywords:** marginal mandibular nerve, anatomage, variation

## Abstract

Anatomage (Anatomage, Inc., San Jose, CA) is a modern method for studying anatomy. It is a state-of-the-art method used for the representation of the structure of the human body. In our study, we examined the seventh cranial nerve of a male Caucasian cadaver using an Anatomage Table in the Anatomy Department of the School of Medicine, National and Kapodistrian University, Athens, Greece. After exiting the skull from the stylomastoid foramen, the facial nerve divided into the temporofacial and cervicofacial main branches. The cervicofacial branch divided into its own branches, including the marginal mandibular nerve (MMN), which ran within the investing (superficial) layer of the deep cervical fascia. We found a variation of the course of the marginal mandibular branch of the facial nerve. In the area of the lower border of the mandible, where the MMN actually crossed the facial artery and vein, it appeared to run deeper than both of those vessels, rather than running superficially. This seemed to be a rare variation of the location of the MMN relative to the facial vessels, which suggested that extra care is essential in surgical approaches within this area.

## Introduction

The anatomy of the human body is a cornerstone of modern medicine. Cadaver dissection has been the exclusive method of studying anatomy for centuries since Vesalius founded modern human anatomy. Complementary atlases of anatomy have always been fundamental to the study of the human body. At first, we generally relied on artists’ drawings. Since then, we have gone through modern color graphics to real pictures taken of cadaver dissections. Such dissections have been the most common method used for educational purposes. However, there are several limitations to this approach. Running an anatomy lab is expensive and time-consuming. Its maintenance and personnel costs are too high, especially considering the constant need for cadaver supply, processing, and conservation. Unfortunately, when performing human cadaver dissection, there is no "undo" or "redo" option. Once a mistake is made and the tissue is inappropriately dissected, there is no going back until the next specimen is available. On the other hand, an increase in the number of students has created a great demand for specimens for training. It is a common practice for an instructor to perform manipulations and dissections while the students watch. Additionally, a cadaver’s structures are fragile and affected by preservative chemicals. When examining a cadaver, visibility is limited to the exposed area of the tissue. In addition, the color and consistency of tissues and organs are quite different in a dead body. Furthermore, rotating a cadaver is not an easy option, meaning usually students can view only a part of the organ or anatomical system being examined. Most of these problems have been solved by the digitizing of human anatomy [1].

The Anatomage method has made great contributions in the attempt to visualize human anatomy. It is based on the concept of a virtual dissection table, reproducing a real cadaver bed and demonstrating real patient data on a real-size scale. It uses the same technology as the CT and MRI. Anatomage presents 3D reconstructions of the human body by combining data of the Visible Korean Project and the Visible Human Project, thus representing a quite accurate digital visualization of human anatomy. These 3D reconstructions can be dissected for internal inspection, and then subsequently segmented and annotated. This creates a safe working environment, free of any chemicals or hazardous materials that might harm students and other personnel during cadaver dissection [2].

In the Visible Human Project, researchers compiled data from MRI, CT, and anatomic images of a 38-year-old (at the time of death) male cadaver. The cadaver underwent CT scanning at 1 mm intervals through the head and neck; 3 mm intervals through the thorax, abdomen, and pelvis; 5 mm intervals through the lower extremities and, several months later, at 1 mm intervals through the entire body. The cadaver was then cut using a cryomacrotome at 1 mm intervals, and the slices were photographed with a digital camera. Cryosections and CT scans were combined with these photos of the slices. The same procedure was performed with the MRI. As part of the same Visible Human Project, the same procedure was carried out with a female cadaver, with anatomical images taken at 0.33 mm intervals [3,4].

In the Visible Korean Project, a Korean male cadaver was chosen without any preservative procedure. The cadaver underwent MRI and CT scanning at 1 mm intervals. It was then frozen in a deep freezer and serially sectioned at 0.2 mm intervals using a cryomacrotome. Each sectioned surface was photographed with a digital camera, and 11 anatomical organs were segmented to produce segmented images. The anatomical and segmented images were perfused and reconstructed to produce 3D images [5,6].

## Case presentation

We studied a Caucasian male specimen of the Anatomage Table’s database (Anatomage Table 5) in the Anatomy Department of the Medical School of the National and Kapodistrian University of Athens. We discovered that the marginal mandibular nerve (MMN) appears as a single branch on both (left and right) sides. After its emergence from the cervicofacial division within the parotid gland, it exited the gland and ran above the lower border of the mandible at a maximum distance of 14.2 mm on the left and 12.8 mm on the right side.

**Figure 1:**
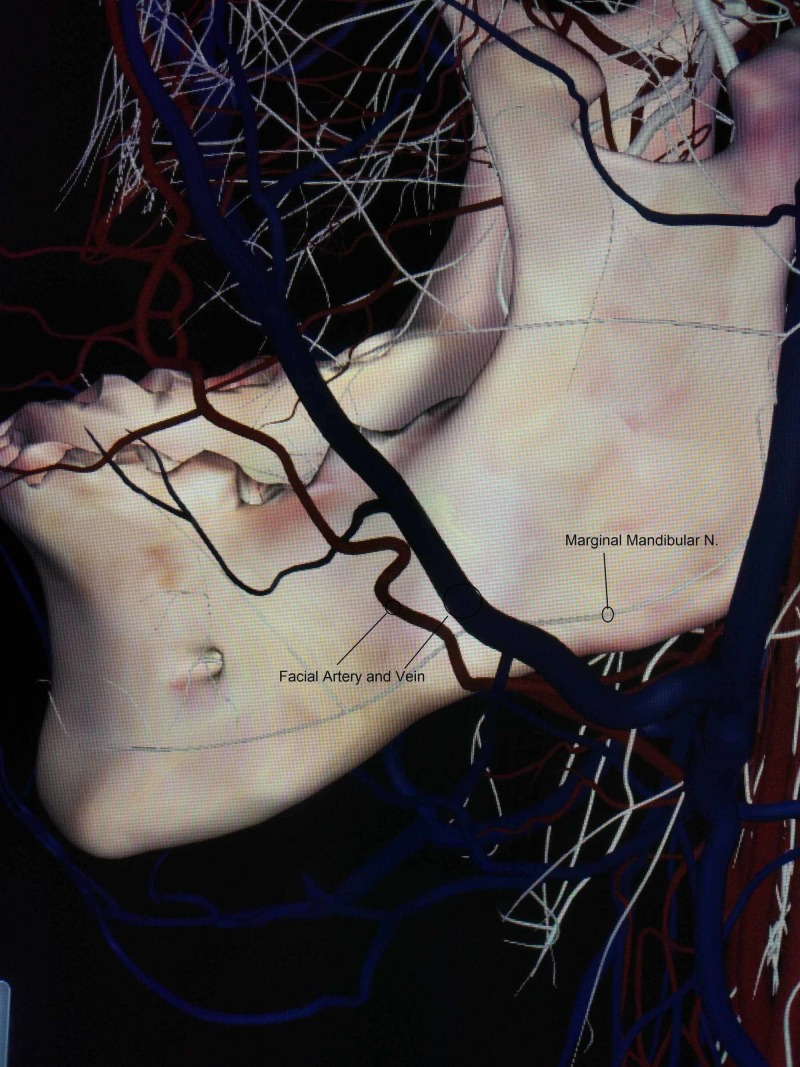
Lateral view of the single branch of the left marginal mandibular nerve, which runs deeper than the facial artery and vein

**Figure 2:**
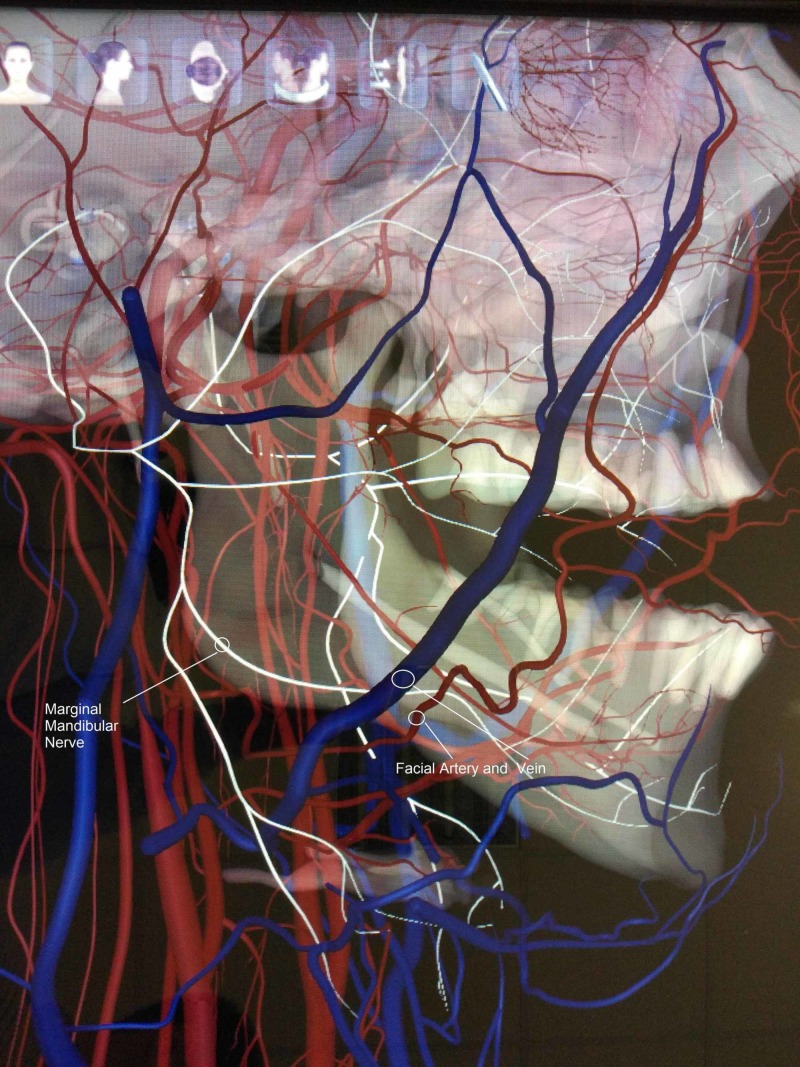
Lateral view of the single branch of the right marginal mandibular nerve, which runs deeper than the facial artery and vein

**Figure 3:**
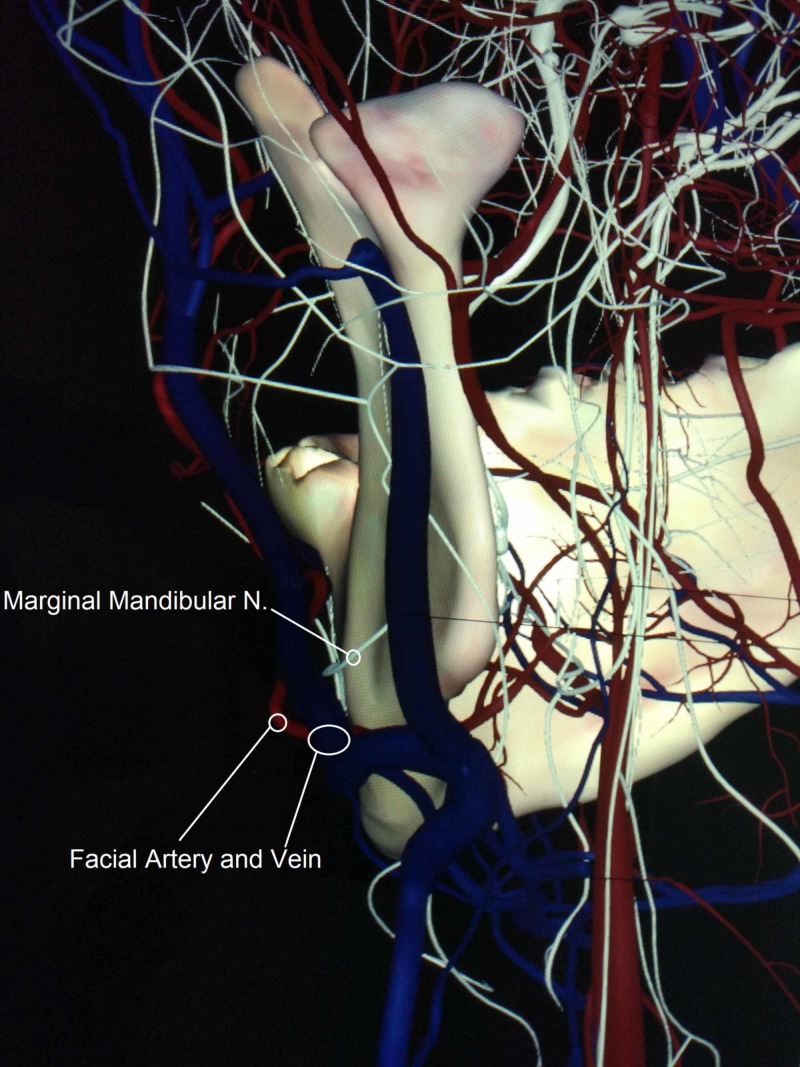
Retro-lateral view of the left marginal mandibular nerve, indicating a single branch that runs between facial vessels and the mandible

We noticed an extremely rare variation in the course of the MMN and its location relative to the facial vessels. It was found deeper (medially) than the facial artery and vein instead of running superficially when crossing them, near the lower border of the mandible, still within the investing layer of the deep cervical fascia on both the right and left sides (Figures 1,2,3).

## Discussion

The facial nerve is the seventh cranial nerve. It controls the facial muscles, gives sensory supply to the anterior two-thirds of the tongue and secretomotor supply to the submandibular, sublingual, and lacrimal glands. After its intracranial course, it exits the skull at the stylomastoid foramen. It then feeds into the posterior auricular branch and the supply branches for the posterior belly of the digastric muscle and the stylohyoid muscle. It then enters the parotid gland. After a short run in the gland, the main trunk splits into two main divisions: the temporofacial and the cervicofacial. These form the parotid plexus, which splits into five main branches: temporal, zygomatic, buccal, marginal mandibular, and cervical.

The marginal mandibular branch of the facial nerve supplies the muscles of the lower lip. It emerges from the inferior-anterior border of the parotid gland into the neck, where it lies deep to the platysma, within or just deep to the investing (superficial) layer of the deep cervical fascia. It then runs, in most cases, superficially to the facial artery and vein near the inferior border of the mandible [7]. According to Gray’s Anatomy, the marginal mandibular branch passes below the lower border of the mandible in 20-50% of the cases, at a maximum distance of 1.2 cm [8]. In 1962, Dingman et al. stated that in 81% of specimens, the marginal mandibular branch posterior to the facial vessels passed above the inferior border of the mandible, and in the other 19% of the cases, it ran at a maximum of 1 cm below the inferior border; however, once it was found above the facial artery, it never curved below the mandible [9]. In 2010, Batra et al. found the marginal mandibular branch traversing along the mandible in 52% of the cases, below it in 32% and above it in 16% of the cases. When it was below the mandible, the distance was measured between 1.1 and 1.6 cm [10]. According to Woltmann et al., in 57.7% of the cases, the nerve passed superior to the inferior border of the mandible [11]. Saylam et al. found that the mandibular branch ran above the inferior border of the mandible in 74% of the cases [12]. Rödel and Lang described that in 66% of the cases, the branch ran below the lower margin of the mandible at a maximum of 1.4 cm [13]. Hwang et al. stated that the average distance between the lowest point of the mandibular branch and the inferior border of the mandible was 5.36 mm at the facial artery point [14].

The marginal mandibular branch of the facial nerve was found by Wang et al. to be a single branch in 32% of the cases and as two or more branches in 68% of the cases [15]. In another study, it was one branch in 28% of the cases, two branches in 52% of the cases, three in 18% of the cases, and four in 2% of the cases [16]. According to Dingman et al., there was a single branch in 21%, two branches in 67%, three branches in 9%, and four major branches in 3% of specimens. However, most researchers agree that the marginal mandibular branch of the facial nerve crosses the superficial surface of the facial vessels in 100% of specimens [9]. In 2012, Balagopal et al. found just one division of MMN in approximately 80% of the patients who underwent neck dissection, two branches in 13%, and three or more branches in 7%. In most of the patients with one branch (60%), it was found crossing the facial artery inferior to the lower border of the mandible. In patients with more than one branch, there was a wide variation in the course of the branches in relation to the lower border of the mandible in crossing the facial artery. They also determined that the mean distance of the branches of the MMN from the lower border of the mandible crossing the facial artery was 1.73 mm below it [17].

These wide measurements among different studies show that there is a gross variation in the course of the MMN, which increases when the neck of the patient is extended and rotated to the contralateral side due to the extension of the neck and traction of the tissue. Hwang's findings of the border-nerve distance suggest that the position of the MMN varies depending on the neck position [14,18].

On the other hand, there are differences between neck dissections performed on preserved cadavers and those performed on live patients. In 2004, Touré et al. stated that the MMN ran laterally to the vessel bundle in 51 out of 54 cases, and it was medially to the facial artery but laterally to the vein in two cases and laterally to the artery and medially to the vein in one case [19]. In 2018, Touré et al. dissected 62 half-heads from cadavers and discovered that the MMN was more consistently lateral to the facial vein than to the facial artery [20].

## Conclusions

These findings corroborate that the male Caucasian cadaveric specimen of the Anatomage Table represents a rare variation of the position of the MMN in the area of the mandible when crossing the facial artery and vein near the lower border of the mandible. It is therefore essential to take extra care in submandibular approaches to the neck and the facial skeleton.
